# A Study on the Effect of Adhesive Cavities on the Scuffing Initiation in a Sliding Contact

**DOI:** 10.3390/ma14154296

**Published:** 2021-07-31

**Authors:** Grzegorz Kaczor, Magdalena Machno

**Affiliations:** Department of Rail Vehicles and Transport, Faculty of Mechanical Engineering, Cracow University of Technology, 31-155 Cracow, Poland; magdalena.machno@pk.edu.pl

**Keywords:** scuffing initiation, adhesion, wear testing, oscillatory motion, non-lubricated conditions

## Abstract

Scuffing is a particularly problematic wear phenomenon in sliding contact that has not yet been fully elucidated. The complicated mechanism of the development of this phenomenon results from the simultaneous influence of many factors. There is a continuous need for new research to gain a deeper understanding of the complex frictional processes that scuffing is. Components such as cams, tappets, piston rings and gears are extremely susceptible to scuffing. The idea of the research on the scuffing wear development is the study of the formation of adhesive cavities as the effects of the destruction of adhesive bonds at various operating parameters. The goal of the presented work is the analysis of the influence of the oscillation frequency on the formation of adhesive cavities leading to scuffing. The tests carried out with the use of S235 steel showed that the adhesive cavities on the surfaces of the tested components appear regardless of the adopted values of the oscillation frequency. The surfaces of the specimen and counter-specimen were analyzed before and after wear tests on the block-on-ring test stand at the different values of the oscillation frequency. The conducted research revealed that the greatest change in the values of the friction coefficient occurs with an increase in frequency from 2 to 5 Hz, and the largest change in the number of scuffing initiating cycles occurs with an increase in the oscillation frequency from 1 to 2 Hz.

## 1. Introduction

The phenomenon of scuffing wear has been the subject of research of scientists for many years, however, due to the simultaneous influence of mechanical, chemical, lubricating performance and thermal factors, it has not been fully explained. Scuffing is a combination of abrasive and adhesive wear, what can be found in the works of some researchers. The authors of work [1] concluded that the changes in the geometric structure of the specimen and counter-specimen surfaces can be caused by wear debris, which plays an important role in the abrasion process. The authors proved that wear debris causes abrasive contamination that changes conformity between the sliding surfaces. 

Scuffing is a particularly problematic wear mechanism occurring on the surface of components operated in sliding contact (directional or oscillating) under limited lubrication conditions. The main parts exposed to scuffing include: injectors, cams, tappets, piston rings, gears and the other parts of the internal combustion engines. 

The working conditions of mentioned machine parts are favorable for the development of scuffing due to the sudden changes in operating temperature, load or relative sliding speed. The combination of these factors results in a breaking down the protective oxide layer and providing direct metal-to-metal contact that initiates an important stage for the scuffing occurrence, which is the local adhesion and the formation of adhesive cavities and the seizure as a final result [2,3]. For this reason, surface scuffing resistance is often equated with the durability of the lubricating film or protective coating. Scientists investigated the possibility of limiting the development of scuffing by applying protective coatings of selected materials, including titanium nitride (TiN) ceramic layers, DLC (diamond-like carbon), TiN, Cr-ceramic and TiAlN, zirconia (ZrO_2_) ceramic, aC: H: W and MoS_2_/Ti coatings [4,5,6].

The use of dedicated research methods is an extremely important factor on the way to a greater understanding of scuffing. For example, the authors of work [7] proposed their own method to test the resistance of coated gears to scuffing. In turn, the authors [8] investigated the possibility of monitoring the surface condition using acoustic emission to enable early detection of scuffing. The conducted research confirmed the high accuracy of the proposed method. A similar approach was used by the authors of work [9] who designed a scuffing observation system and registered a plastic flow in the contact area.

A list of selected proposals for scuffing wear definitions can be found, among others in work [10]. One definition specifies scuffing as a local damage to associated surfaces in sliding motion, caused by welding solids without local melting of the contact surface. According to another definition, scuffing is considered to be surface a damage in sliding motion caused by the formation of local welds. Scuffing may be also identified as the process of dulling the surface, caused by plastic flow, which may be accompanied by loss of material or its transfer.

Catastrophic wear occurring in the case of boundary lubrication occurs in various combinations of friction pairs, which was proved by the authors of the work [11] who conducted tests on the Four Ball Machine and Pin-on-disk Machine stands.

Despite numerous studies on scuffing, there are some discrepancies in the causes and development of this type of wear. Among the available scientific studies, based on experimental tests, it has been established that the occurrence of scuffing is preceded by the rapid changes in the friction coefficient, temperature on the contact surfaces as well as the formation of excessive vibrations and noise [12,13,14]. However, the authors of work [15] noted induced material removal caused by scuffing as well as the fatigue cracks in the areas of the worn surfaces. In turn, Galligan, Torrance and Liraut [7] noticed that the rapid increase in friction on the surfaces of the friction couple may result from the increased surface smoothness and damage of the lubricating film layer.

A lot of research concerns parts of internal combustion engines and gears [16,17,18]. C-Lorentzo Martin et al. [19] evaluated scuffing performance of Y_2_O_3_-stabilized ZrO_2_ ceramic material which may be used in fuel injector systems. C. Zhang et al. [20] developed a model to predict scuffing initiation based on the adiabatic shear instability phenomena. They found out that the temperature of friction work is greater than temperature caused by a plastic deformation work. In turn, the authors of work [21] examined the effect of Oil Free Water-Based Lubricant on scuffing wear resistance in gear application using the selected FZG (Forschungsstelle für Zahnräder und Getriebebau, also called Gear Research Centre) scuffing test methods. They showed the possibility of increasing the scuffing load depending on the kind of plant extract. The authors of the work [22] showed that the operating parameters of the gears, such as temperature and lubrication behavior may affect the meshing losses. A scuffing behavior in a cylinder liner with different micro-dimpled textures was the subject of the study [23]. They indicated the influence of the selected geometric parameters (diameter, area fraction) of a micro-dimple on the friction coefficient. Review of the gear methods for tooth fault detection using vibration analysis is included in the paper [24].

Scuffing wear tests are not limited to experimental testing only. Much of the research work is devoted to mathematical models that can detect scuffing before surface damage occurs. These are, among others: temperature models, models based on elastohydrodynamic lubrication (EHL), desorption models, lubricant decomposition models, asperity interaction models, film formation and removal models. Bowman and Stachowiak [10] provided an extensive review of these models. Some researchers use a combination of several models to study scuffing criteria. The authors of [25] developed the elastohydrodynamic lubrication (EHL) model combined with a heat transfer model in order to enable the prediction of changes in the friction coefficient, the sudden increase of which is one of the symptoms of scuffing. Advanced mathematical models take into account also the plastic deformation, the type of material and specific contact conditions in sliding contact. These are the most common models based on adiabatic shear instability [26,27].

As part of the analytical research, the friction characteristics, temperature characteristics of the specimen and counter-specimen in the area of contact against sliding cycles were developed. Sudden increases in the friction coefficient value for different sets of input parameters were captured. The similar approach was used by other researchers [8,28].

Since the scuffing wear process depends on the influence of many factors at the same time, and the occurrence of sudden increases in the friction coefficient values is random, the development of the scuffing wear mechanism is not a simple task. Therefore, assuming an appropriate research model that takes into account all the phenomena associated with scuffing is a challenge. There is no doubt that each additional empirical study represents an important next step towards a better understanding of scuffing. According to the above description, the further experimental research is needed to understand the scuffing phenomena.

In the article, the experimental part of research includes the observation and identification of the abrasive and adhesive wear as the elementary scuffing processes on the worn surfaces. Numerous cavities were identified on the worn surface of the specimen, as the evidence of the adhesion occurrence.

The following paper investigates the oscillation frequency on the scuffing wear for a selected steel grade under non-lubricated conditions. The study was preceded by the review of the literature on scuffing wear and the performance of the numerous preliminary tests, which allowed to determine the test conditions and the range of the input parameters: the contact force *F*, the oscillation angle *α* and the oscillation frequency *f*. The applied research approach is based on the method proposed in scientific articles for the detection of scuffing initiation by sudden changes in the friction coefficient. The added scientific value of this work consists in a thorough examination of the phenomenon of adhesive cavities formation at various operational parameters of the friction pair.

## 2. Materials and Methods

In the following research, the wear tests were performed with the use of specimen and counter-specimen made from S235 steel. This is a commonly used material in the production of components such as rings, shafts and pins. Selected mechanical properties of S235 steel are presented in the Table 1.

**Figure 1:**
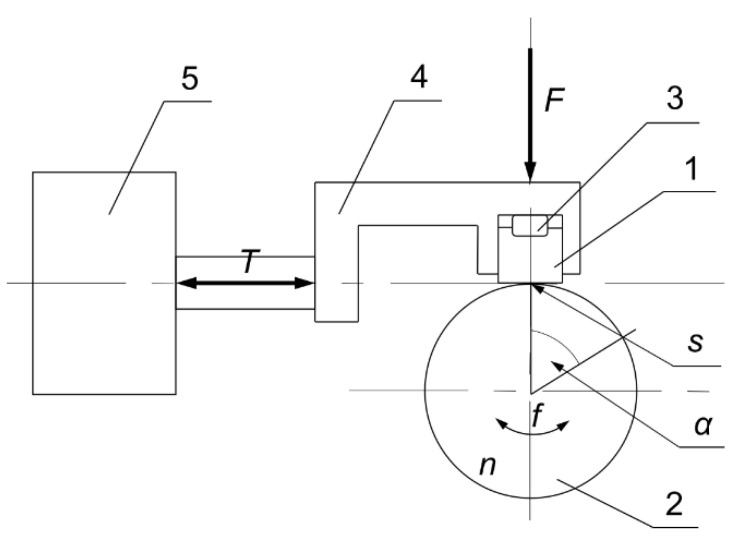
Scheme of the test stand (1—specimen, 2—counter-specimen, 3—semicircular canopy, 4—holder, 5—force sensor [29].

Taking into account the above conditions, it was decided to deliberately use steel type S235 as both the specimen and counter-specimen material, because the use of homonymous materials increases the risk of adhesion occurrence. The wear tests were performed only under non-lubricated friction conditions to induce the adhesion. Thanks to the use of the same type of sample and counter-sample material—S235 steel and non-lubricated conditions, favorable conditions were ensured for the rapid achievement and development of the adhesion phenomenon, which was an intentional action. A T-05 block-on-ring test machine (Łukasiewicz-ITeE, Radom, Poland) was used to carry out the experiments. The kinematic scheme of the friction joint is shown in Figure 1.

**Figure 2:**
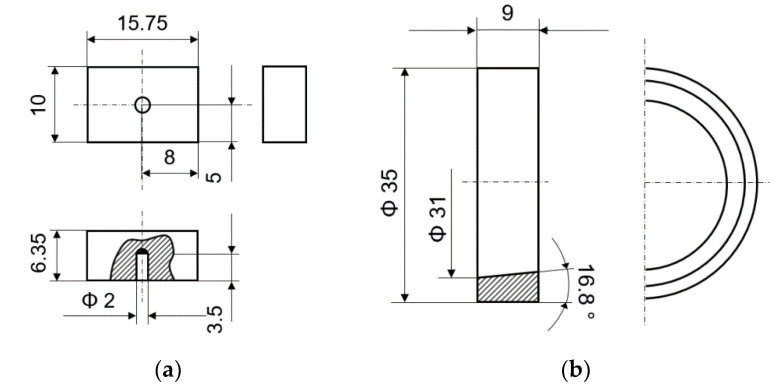
The dimensions of: (**a**) specimen; (**b**) counter-specimen [29] (Unit: mm).

The test machine allows to conduct the experiments at different values of the contact force (*F*), sliding velocity (*n*), friction area (*s*), frequency (*f*) and angle (*α*) of oscillation. The block used as a specimen is fixed in the holder by a semicircular canopy. The counter-specimen, embedded in a shaft, makes a move with a given speed in one direction or makes an oscillating motion with an appropriate value of the frequency and angle. The dimensions in millimeters for the specimen and counter-specimen are shown in Figure 2. Moreover the temperature of the friction pair components was measured by the thermocouples. The measuring tips were placed at the measuring points of the test stand according to the original documentation of the T-05 test stand.

The experimental tests were carried out in the room temperature under constant values of the contact force and the oscillation angle: *F* = 0.1 N and *α* = 10°. The variable input was the oscillation frequency f, which values were changed during the following values: 1.0, 2.0, 5.0, 7.5 and 10.0 Hz, respectively.

For each set of parameters (*F*, *α*, *f*) three tests were performed, bringing the total number of 15 tests. The first test for a given set was carried out for a fixed number of oscillation cycles equal to 10,000. The other two tests were performed until a sudden friction coefficient increase occurred, which is one of the symptoms of scuffing wear. After a sudden increase in the value of the friction coefficient, such tests were terminated and the surfaces in the wear area were observed. The assumption of the selected values of input parameters was preceded by the preliminary tests conducted by the authors as well as the results of previous studies made by other researchers, as mentioned earlier. This approach to scuffing wear tests was also used in the works [3,28,30]. During the tests, the friction force as well as the temperature in the contact area were measured.

The experimental conditions used in the presented paper were selected in such a way as to maximize the probability of occurrence of scuffing wear. On the basis of the research results and guidelines presented in [7,12,31], the following conditions for the scuffing occurrence can be assumed:

- combining the components of the friction pair in the oscillating or directional motion,
- leading to direct metal-metal contact,
- the occurrence of adhesive wear,
- the occurrence of abrasive wear.

**Figure 3:**
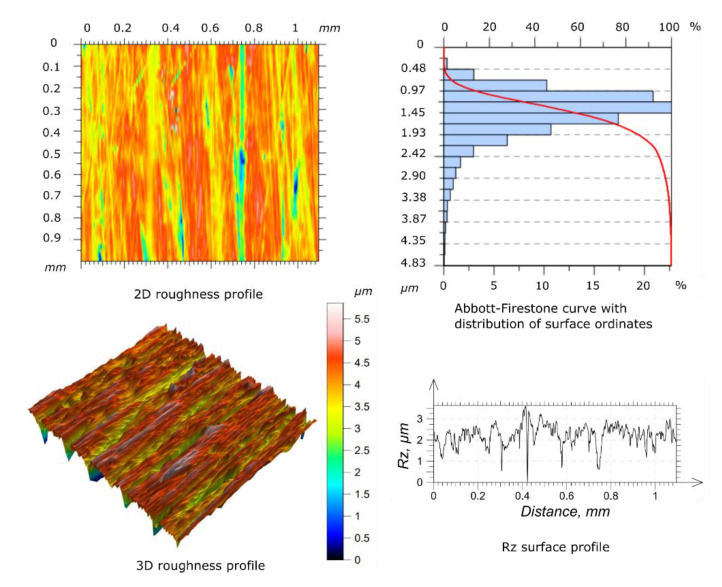
Geometric structure of the surface before the wear tests for specimen.

**Figure 4:**
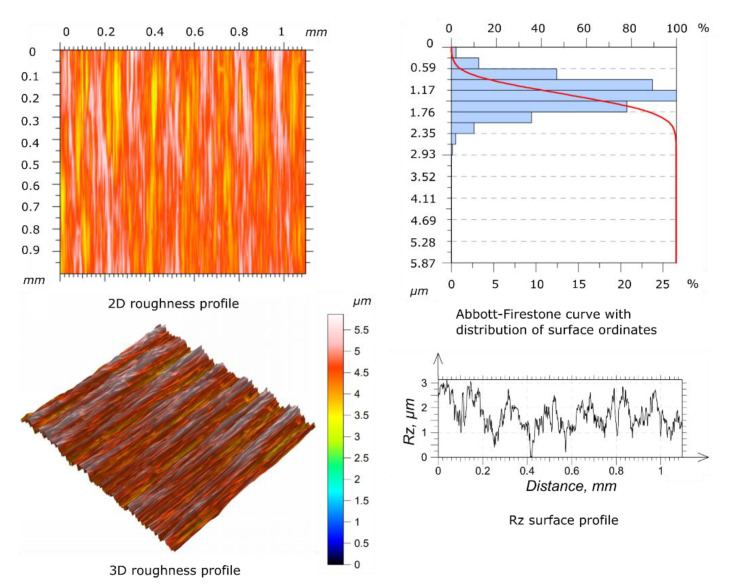
Geometric structure of the surface before the wear tests for counter-specimen.

Before starting the wear tests, the surface roughness measures of the specimen and counter-specimen were conducted, according to the requirements of ISO 4287 [32] standard. To perform the measures, the authors used a Taylorsurf Intra 50 profilometer (Taylor Hobson, Leicester, UK), equipped with a rounding radius measuring tip of 2 µm. The surface roughness of the tested specimens and counter-specimens was measured three times and the average result was taken into account as a reference (mean values of *Ra* and *Rz*). The surfaces of the specimen and counter-specimen were processed by grinding in order to obtain a similar geometric structure. The results of the surface roughness for the reference specimen and counter-specimen are shown in the Figure 3 and Figure 4, respectively.

The average parameters of surface roughness for the basic specimen and counter-specimen before the wear tests are included in Table 2.

**Figure 5:**
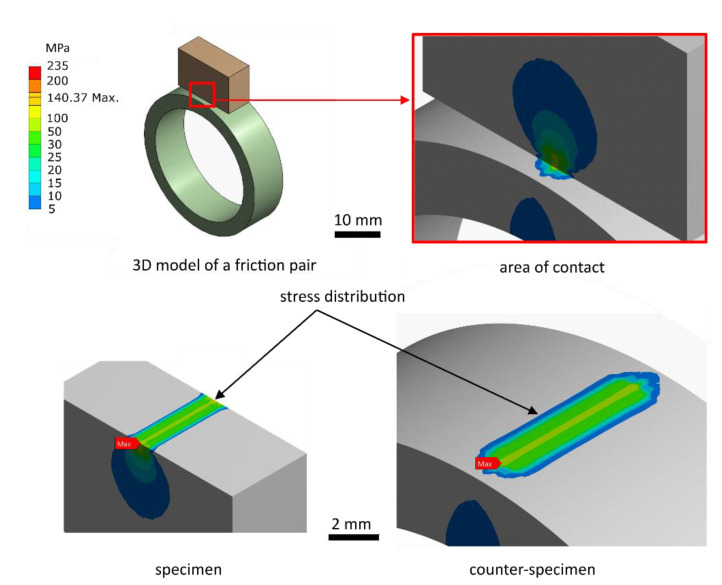
Stress distribution for the tested specimen and counter-specimen.

Before starting the actual tests, a static analysis of the stress distribution in the contact area of the specimen and counter-specimen was performed. The aim was to show that the maximum stress value in the contact area does not exceed the yield point of the materials of the friction pair components (Table 1). The reduced stress distribution was obtained by the Huber-Mises-Hencky (H-M-H) method on the friction pair model made in the SolidWorks environment (Figure 5). The second-order cubic finite element type was used in the mesh. The element size was selected to obtain at least 4 finite elements for the counter-specimen thickness, which is the smallest dimension in the entire friction pair model. The value of 300 MPa for the maximum pressure was purposefully adopted, which was not used during the research. The main aim was to prove the lack of plastic deformation during the cooperation of the friction pair.

Based on the stress state analysis, it can be concluded that the yield strength of S235 steel was not exceeded for the applied pressure force. It will not be exceeded, the more so for the pressure force applied in the wear tests, *F* = 0.1 N. Therefore, the influence of plastic deformation on the disturbance of scuffing wear tests is considered negligible.

## 3. Results, Analysis and Discussion

The following chapter includes the results analysis of the investigation on the influence of the oscillation frequency on the development of scuffing wear in the block-on-ring model under non-lubricated conditions.

### 3.1. Analysis of the Results of Wear and Temperature Measurements in the Contact Area

**Figure 6:**
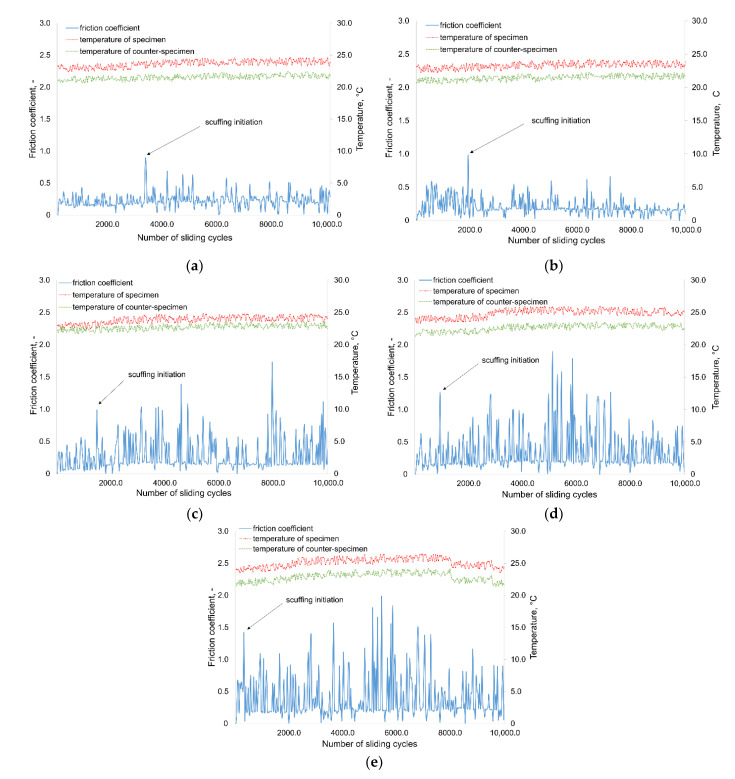
Results of the friction coefficient and temperature measurement for the example set: *F* = 0.1 N, *α* = 10°, *f* = 1 Hz and various value of f: (**a**) 1.0 Hz; (**b**) 2.0 Hz; (**c**) 5.0 Hz; (**d**) 7.5 H; (**e**) 10.0 Hz.

The characteristics of the coefficient of friction and the temperature of the specimen and counter-specimen with the contact area for the selected set of parameters are presented in Figure 6. There is a sudden increases in the value of the friction coefficient, which correspond to the relevant numbers of sliding cycles. This sudden increase in the value of the friction coefficient is one of the symptoms of scuffing wear, which was also used by researchers in other studies [3]. The values of the friction coefficient and the number of sliding cycles at which a significant increase of friction occurred clearly changed depending on the value of the oscillation frequency. As can be seen from the friction coefficient graphs in Figure 6, the higher the oscillation frequency, the earlier the scuffing wear begins as evidenced by the sudden increases in the friction coefficient. This regularity was observed for all the tests performed. The temperature values of the specimen and the counter-specimen in the contact varied between 21 and 27 Celsius degrees as a result of increasing friction.

**Figure 7:**
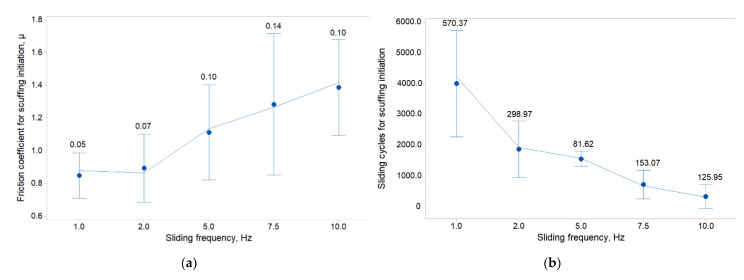
Interval plots of the scuffing initiation, related to: (**a**) friction coefficient; (**b**) sliding cycles.

On the basis of the compared mean values of the operating cycles at which the scuffing initiates, it can be concluded that for higher oscillation frequency values, the wear process at the point of contact between specimen and counter-specimen is faster and scuffing occurs earlier (Figure 7b).

The impact of the oscillation frequency on the value of the friction coefficient at which scuffing initiate is presented in Figure 7a. In the range of the considered values of the oscillation frequency, it can be seen that for the values of *f* = 1 and 2 Hz there is only a slight difference (about 10%) in the values of the friction coefficient at which scuffing starts to occur. By increasing the oscillation frequency from 2 to 5 Hz, we increase the friction coefficient value (about 43%), at which scuffing is initiated. The difference between the value of the friction coefficient initiating scuffing for the smallest and the largest of the adopted oscillation frequency values is 100%. An approximately proportional increase in the mean value of the friction coefficient initiating scuffing can be seen when increasing the value of the oscillation frequency in the range from 5 to 10 Hz. It can also be observed that the standard deviation from the mean value of the friction coefficient increases with increasing oscillation frequency, except for the last tested value, *f* = 10 Hz. In oscillatory motion, the occurrence of scuffing is more favorable than in unidirectional motion, and the additional increase in the oscillation frequency is a factor which is additionally unfavorable for the value of the friction coefficient at which scuffing is initiated [12].

The relationship between the sliding cycles for scuffing initiation and the sliding frequency is shown in Figure 7b. In the case of this relationship, the opposite is true to the effect of the oscillation frequency on the value of the friction coefficient that initiates scuffing. As the frequency of the oscillation increases, the number of cycles required for a sudden increase in the friction coefficient to occur decreases. This is due to the fact that with a higher value of the friction frequency, the wear processes occur faster. To mitigate the violent effects of scuffing, it is recommended to avoid high oscillation frequencies during operation of friction pair. The largest difference in the change in the average number of cycles for scuffing initiation is noticeable with increasing oscillation frequency of 1 to 2 Hz (about 65%). The smallest difference in sliding cycles for scuffing initiation (about 366%) occurs when the oscillation frequency is changed from 2 to 5 Hz. The difference in sliding cycles for scuffing initiation between the smallest and the largest of the applied oscillation frequencies is about 466%. The higher the value of the oscillation frequency, the faster the adhesive process occur, generating smaller deviation of the results around the mean value.

### 3.2. Results Analysis of Worn Surfaces in the Contact Area

**Figure 8:**
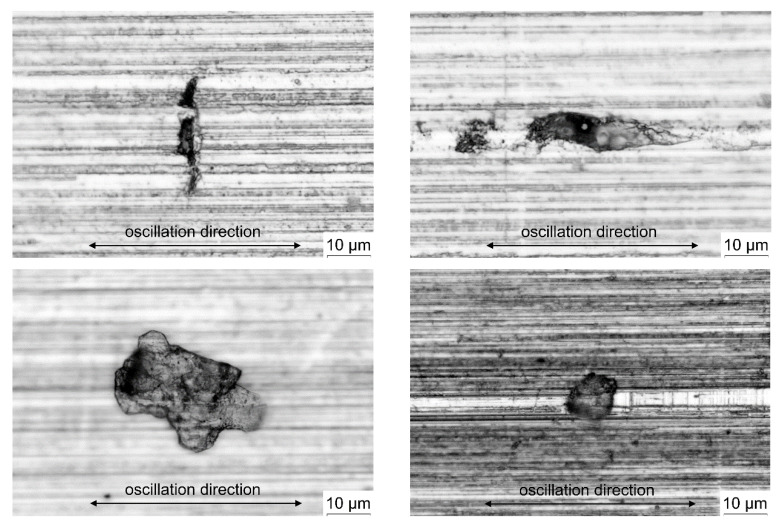
The examples of adhesive cavities observed after the scuffing tests.

The analysis of the worn surfaces of the friction pair components is based on the observation with the use of microscopic photography, roughness profile measurement and the assessment of changes in the geometric structure. The results of the observation of the surface of the example worn specimens are shown in Figure 8. These are the photos taken after the test was stopped, caused by a sudden increase in the friction coefficient value. There are visible local damages to the surface, called adhesive cavities.

**Figure 9:**
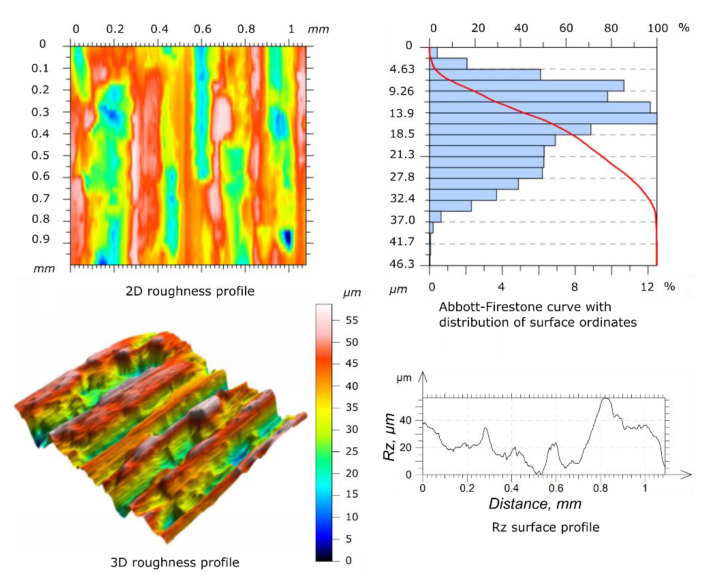
Geometric structure of the surface after the wear tests for specimen (conditions of test: *F* = 0.1 N, *α* = 10°, *f* = 5 Hz).

**Figure 10:**
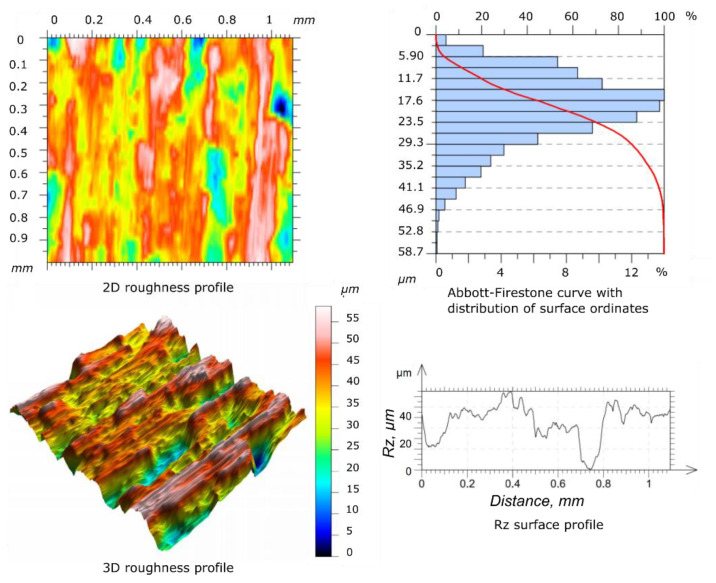
Geometric structure of the surface after the wear tests for counter-specimen (conditions of test: *F* = 0.1 N, *α* = 10°, *f* = 5 Hz).

The study of the geometric structure of the surface of the specimen and the counter-specimen after wear tests showed significant differences in the parameters of the roughness profile in relation to the base surfaces, as shown in Figure 9 and Figure 10, respectively. The values of the *Ra* and *Rz* parameters increased with the increase of the oscillation frequency values applied. The shape of the Abbott-Firestone curve can be inferred from the resistance of a given surface to abrasive wear. The progressive curve means that the analyzed surface does not have “sharp” peaks of micro-roughness and is more resistant to abrasive wear than surfaces with uniform course of this curve.

To present the geometric structure after the wear process, only selected results of the surface roughness profile measurement are showed.

Table 3 shows the measurement results of the *Ra* and *Rz* parameters for the surface of the selected specimen and the counter-specimen after wear tests, which were terminated after the first significant increase in the friction coefficient value. It can be seen that the values of the *Ra* and *Rz* parameters for the specimen and the counter-specimen changed significantly after testing. The *Ra* value for specimen and the counter-specimen increased by more than 17 and 12 times, respectively. The same applies to the *Rz* parameter, which increased by more than 9 and 6 times for the specimen and the counter-specimen, respectively. This is due to the lack of a lubricant between the cooperating surfaces, as a result of which the adhesive and abrasive wear processes led to the transition of the technological surface layer into the operational layer. Using this approach, the scuffing phenomenon is captured in a shorter time, and the influence of the lubricant on the physical development of the phenomenon is negligible.

The conducted research on scuffing revealed that in the adopted range of the tested parameters, there are conditions favorable for the occurrence of adhesion, which results in losses on the surface of the assumed steel type S235.

The obtained results of the analytical tests showed that the change of the oscillation frequency does not always have a significant influence on the value of the friction coefficient and the number of operating cycles at which scuffing is initiated. In the case of investigating the influence of the oscillation frequency on the value of the friction coefficient, the greatest change in the mean value of the friction coefficient occurs when the oscillation frequency is increased from 2 to 5 Hz. In the case of analyzing the influence of the oscillation frequency on the number of operating cycles, the most important change is the initial oscillation frequency (from 1 to 2 Hz).

In the initial stage of cooperation of the friction pair components, during non-lubricated metal-metal contact, the surfaces of the specimen and counter-specimen are smoothed, related to their lapping. The actual contact area is increased, which in the case of materials with identical mechanical properties leads to the creation of conditions for the occurrence of adhesion. Then a sudden increase in the value of the friction coefficient is observed, which may indicate the development of a certain resistance to motion during the cooperation of the friction pair, caused by the formation and bonds. Continued cooperation of the components of the friction pair leads to the destruction of the adhesive bonds, which leaves extensive cavities. Material debris is displaced in the friction joint, resulting in an enlarged area of surface damage. This is confirmed by microscopic observation of the surfaces of the tested specimens. The unanimity of the materials of the cooperating surfaces favors the formation of subsequent adhesive bonds, leading to plastic deformation of the roughness asperities. This confirms the approach expressed in [26] that scuffing is initiated precisely by local plastic deformations in the micro-contact areas, caused by the phenomenon of adhesion. On the other hand, in another study it can be found that scuffing takes place only when adhesive wear products appear in the friction node, which are detached particles of a weaker material [14]. The reasons for the formation of cavities should therefore be sought in the consequences of plastic deformations of the roughness asperities. In the next stage of cooperation of the surfaces of the friction pair, the progressive development of adhesive cavities takes place, which creates conditions for the spread of cavities and growths into the surface layer.

## 4. Conclusions

The reduction of scuffing wear in the oscillating motion in non-lubrication conditions can be achieved to a greater extent by appropriate selection of the operating parameters. Therefore, the appropriate selection of the values of oscillation frequency may also complement other methods of reducing the occurrence of scuffing (selection of appropriate lubricants, shaping the properties of the surface layer), when non-lubricated friction conditions occur. The most important conclusions obtained from the conducted research includes:

1. The cavities appearing on the surfaces of the tested components occur regardless of the applied value of the oscillation frequency (within the accepted range).
2. The effect of the oscillation frequency on the value of the friction coefficient and the number of operating cycles at which scuffing is initiated is significant only for selected frequency intervals (from 2 to 5 Hz in case of friction coefficient, from 1 to 2 Hz in case of operating cycles).
3. The occurring symptoms of scuffing (sudden increase in the friction coefficient values and adhesive cavities on the worn surfaces) are independent of the oscillation frequency values adopted for the study.
4. By increasing the oscillation frequency, the deviation of the friction coefficient values initiating scuffing increases, while the number if operating cycles at which scuffing symptoms are observed decreases (with little exceptions).

The scope of the research carried out included the analysis of the influence of selected operating parameters on the development of scuffing wear. It is necessary to continue the conducted research to fully explain the development of scuffing wear. More extensive research and measurement of worn surfaces are planned using modern equipment (e.g., SRV methods and non-contact methods of surface roughness measurements). Future research should take into account a different range of the input parameters, other specimen and counter-specimen materials and their surface treatment.

## Figures and Tables

**Table 1:** Basic mechanical properties of the S235 steel [29].

Designation	Value
Yield strength Re, MPa	235
Tensile strength Rm, MPa	410
Brinell Hardness HB, -	140
Relative elongation A_5_, %	21–24

**Table 2:** Average roughness parameters of surfaces on the area of contact for specimen and counter-specimen before the wear test.

Designation	Specimen	Counter-Specimen
*Ra*, µm	0.37	0.43
*Rz*, µm	3.52	2.61

**Table 3:** Average roughness parameters of surfaces on the area of contact for specimen and counter-specimen after the wear test.

Designation	Specimen	Counter-Specimen
*Ra*, µm	6.40	5.20
*Rz,* µm	37.20	30.50

## Data Availability

The data presented in this study are available on request from the corresponding author. The data are not publicly available due to privacy.
